# Characterization of prognostic value and immunological roles of RAB22A in hepatocellular carcinoma

**DOI:** 10.3389/fimmu.2023.1086342

**Published:** 2023-03-03

**Authors:** Fukai Wen, Fanshuai Meng, Xuewen Li, Qingyu Li, Jiaming Liu, Rui Zhang, Yunzheng Zhao, Yu Zhang, Xin Wang, Shuai Ju, Yifeng Cui, Zhaoyang Lu

**Affiliations:** ^1^ Department of Hepatic Surgery, The First Affiliated Hospital of Harbin Medical University, Harbin, China; ^2^ Key Laboratory of Hepatosplenic Surgery, Ministry of Education, The First Affiliated Hospital of Harbin Medical University, Harbin, China; ^3^ The Department of Inpatient Central Operating Room, The First Affiliated Hospital of Harbin Medical University, Harbin, China; ^4^ Department of Respiratory and Critical Care Medicine, The First Affiliated Hospital of Harbin Medical University, Harbin, China

**Keywords:** RAB22A, hepatocellular carcinoma, cancer immune infiltrates, prognosis, biomarker, bioinformatics analysis

## Abstract

**Background:**

The protein-coding gene *RAB22A*, a member of the RAS oncogene family, is amplified or overexpressed in certain cancers. However, its action mechanism in hepatocellular carcinoma (HCC) remains unclear. Here, we aimed to examine the connection between *RAB22A* and survival prognosis in HCC and explore the biological significance of RAB22A.

**Methods:**

A database-based pan-cancer expression analysis of RAB22A was performed. Kaplan–Meier analysis and Cox regression were performed to evaluate the association between *RAB22A* expression and survival prognosis in HCC. Using Gene Ontology (GO), Kyoto Encyclopedia of Genes and Genomes (KEGG), and Gene Set Enrichment Analysis (GSEA), various potential biological functions and regulatory pathways of RAB22A in HCC were discovered. Tumor immune infiltration was studied using the single sample gene set enrichment analysis (ssGSEA) method. N6-methyladenosine modifications and the regulatory network of competitive endogenous RNA (ceRNA) were verified in the TCGA cohort.

**Results:**

*RAB22A* was upregulated in HCC samples and cell lines. A high *RAB22A* expression in HCC was strongly correlated with sex, race, age, weight, TNM stage, pathological stage, tumor status, histologic grade, TP53 mutation status, and alpha fetal protein (AFP) levels. Overexpression of *RAB22A* indicated a poor prognosis was related to overall survival (OS), disease-specific survival (DSS), and progression-free interval (PFI). GO and KEGG analyses revealed that the differentially expressed genes related to RAB22A might be involved in the proteasomal protein catabolic process, ncRNA processing, ribosome ribosomal subunit, protein serine/threonine kinase activity, protein serine kinase activity, Endocytosis, and non-alcoholic fatty liver disease. GSEA analyses revealed that the differentially expressed genes related to RAB22A might be involved in the T cell receptor, a co-translational protein, that binds to the membrane, axon guidance, ribosome, phagocytosis, and Eukaryotic translation initiation. *RAB22A* was correlated with N6-methyladenosine expression in HCC and established *RAB22A*-related ceRNA regulatory networks. Finally,*RAB22A* expression was positively connected the levels of infiltrating with T helper cells, Tcm cells, and Th2 cells,In contrast, we observed negatively correlations with cytotoxic cells, DCs, and pDCs cells.Moreover,*RAB22A* expression showed a strong correlation with various immunomarkergroups in HCC.

**Conclusions:**

RAB22A is a potential therapeutic target for improving HCC prognosis and is closely related to immune cell infiltration.

## 1 Introduction

Hepatocellular carcinoma (HCC) is the sixth most diagnosed cancer and the fourth leading cause of cancer death worldwide, with approximately 841,000 new cases and 782,000 deaths annually (1). Many key factors, including infection with hepatitis B or C and contact with foods contaminated with aflatoxin, contribute to HCC development (2). Surgery is the typical treatment for HCC; however, the disease is prone to relapse and metastasis, making it difficult to cure (3). Therefore, there is an urgent need to identify new relevant biomarkers to improve the early diagnosis, prognostic assessment, and treatment of HCC.

RAB22A is a small GTPase that belongs to the RAB protein family, specifically, the RAB5 subfamily (4). This protein is mainly located in early endosomes, Golgi bodies, and late endosomes. RAB proteins are involved in the regulation of vesicular traffic and exosome formation (5). Studies have found that the RAB5 subfamily (including RAB5, RAB21, RAB22A, and RAB22B) is primarily involved in the endocytosis, transport, and metabolism of growth factor receptors and may thus be associated with cancer progression (6–8). *RAB22A* expression is elevated in several malignancies, including breast, colorectal, and osteosarcoma cancer (9–11). It accelerates the progression of malignant tumors *via* various mechanisms, for instance, miRNA downregulation (11), recycling of extracellular matrix metalloproteinase inducer (EMMPRIN) (12), and hypoxia-inducible factor (13). Nevertheless, the function of *RAB22A* in HCC remains unclear.

Furthermore, RAB22A has multiple immune functions and is a novel immunomodulatory factor. Accurate intracellular transport of MHC-I molecules in dendritic cells (DCs) and T lymphocytes depends on RAB22A function (14). RAB22A is also part of the accommodative immune response and is absorbed by a process that separates it from the envelope proteins and spreads it throughout the body (15). Previous research has identified RAB22A as the main endosomal target in pathogen infection and a critical regulator of microbial infection and intracellular transport (16). In summary, *RAB22A* may have a significant prognostic and immunological significance in HCC.

In the current study, we analyzed the expression of *RAB22A* in HCC and paracancerous tissues using multiple datasets and *in vitro* experiments. Additionally, we examined the connection between *RAB22A* and survival prognosis in HCC and explored the biological significance of RAB22A by performing enrichment and protein-protein interaction (PPI) network analyses and determining the correlation with immune cell infiltration. Furthermore, we constructed ceRNA regulatory networks involving RAB22A in HCC. Our study proposes a possible connection between *RAB22A* expression and the presence of immune infiltrates in HCC.

## 2 Materials and methods

### 2.1 Database source and processing

Gene expression and clinical data were extracted from multiple databases (**Supplementary Table 1**) and RAB22A expression levels from RNA-seq data (TPM) of patients with HCC were analyzed. The **Supplementary Materials and Methods** (17–19) presents detailed information on the included data.

### 2.2 Patients and clinical samples

The First Affiliated Hospital of Harbin Medical University provided 30 matched sets of HCC and nearby non-tumor liver samples from patients undergoing hepatectomy between February 2020 and June 2022. This project was approved by the First Affiliated Hospital of Harbin Medical University’s Ethics Committee.

### 2.3 Western blotting and quantitative real-time PCR

Total proteins and total RNA were extracted from HCC samples. Details of the experimental procedures are provided in the **Supplementary Materials and Methods**.

### 2.4 Tumor immune infiltration analysis

We used the single sample gene set enrichment analysis (ssGSEA) method (20, 21) and TIMER database (22, 23) to investigate the relationships between *RAB22A* expression and immune cell infiltration, as detailed in the **Supplementary Materials and Methods**.

### 2.5 Gene set enrichment analysis

Enrichment analyses of relevant functional pathways were performed using the GO and KEGG databases (**Supplementary Tables 3**, **4**) and GSEA (**Supplementary Tables 5**, **6**), as detailed in the **Supplementary Materials and Methods** (24, 25).

### 2.6 Prediction and construction of ceRNA networks

Multiple databases were used to predict and screen the lncRNA-miRNA-mRNA (RAB22A) ceRNA network online. Details are provided in the **Supplementary Materials and Methods**.

### 2.7 Protein interaction network and module analysis

We created the protein–protein interaction (PPI) network using the Search Tool for the Retrieval of Interacting Genes (STRING) database (**Supplementary Table 6**) (26, 27), as detailed in the **Supplementary Materials and Methods**.

### 2.8 Statistical analysis

The R package (version 3.6.3) was used for statistical analyses and plotting. RAB22A expression in unpaired and paired samples was analyzed using the Wilcoxon rank sum test, and Wilcoxon signed rank test, respectively, with the pROC (1.17.0.1) package for ROC analysis. The *RAB22A* expression level was analyzed by querying the GEO, TIMER, and UALCAN databases (18). Using the KM method and log-rank test, we compared the differences in 10-year OS, DSS, and PFI between patients with high RAB22A expression and those with low RAB22A expression in TCGA. Cox analysis was used to determine the correlation between *RAB22A* expression and clinical features. *p* < 0.05 was considered to indicate significance.

## 3 Results

### 3.1 *RAB22A* is upregulated in HCC

**Figure 1:**
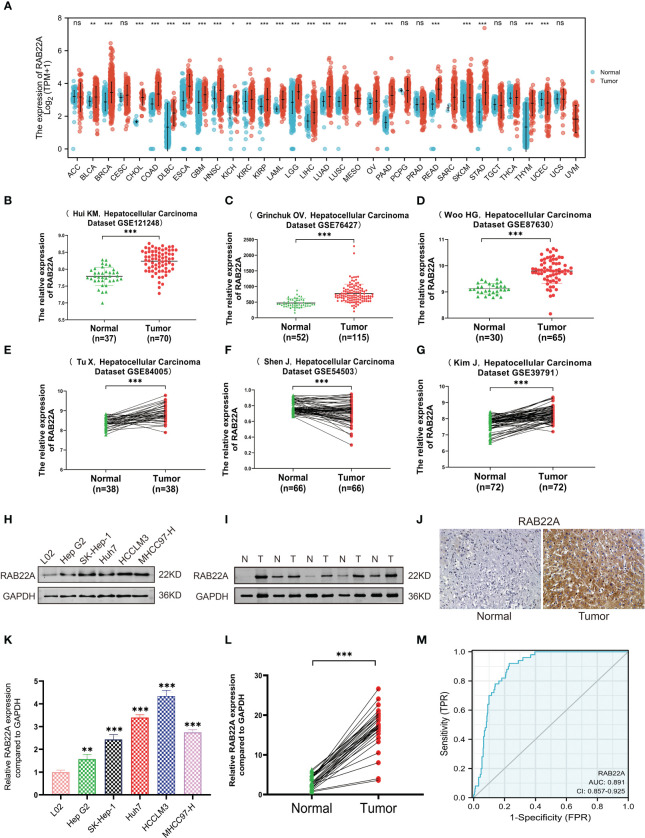
Expression level of *RAB22A* in HCC was verified in TCGA and GEO databases and *in vitro* experiments. **(A)** Comparison of the expression levels of *RAB22A* in different cancerous and normal tissues. **(B–G)** CEO database analysis of *RAB22A* expression in HCC tissues. **(H)** Western blotting assay of RAB22A protein expression levels in L02, Hep G2, SK-Hep1, Huh7, HCCLM3, and MHCC97-H cell lines. **(I)** Western blotting assay of *RAB22A* protein expression levels in HCC and adjacent tissues. **(J)** *RAB22A* protein levels in normal liver and HCC were measured using IHC. **(K)** qRT-PCR assay of RAB22A mRNA expression levels in L02, Hep G2, SK-Hep1, Huh7, HCCLM3, and HCCH97-H cell lines. **(L)** qRT-PCR assay of *RAB22A* mRNA expression levels in 30 pairs of HCC and adjacent tissues. **(M)** ROC curves were created to investigate the value of *RAB22A* in identifying HCC tissues. *p < 0.05, **p < 0.01, ***p < 0.001, NS, no significance.

First, we examined the *RAB22A* expression levels in different malignancies by assessing TCGA databases. RAB22A was highly expressed in 33 malignant tumors, including HCC (**Figure 1A**). In addition, *RAB22A* was highly expressed in the GEO datasets GSE121248, GSE87630, GSE76427, GSE84005, GSE57957, and GSE39791 HCC samples (*p* < 0.001) (**Figures 1B–G**). Western blot analysis of human normal liver cells (L02) and HCC cells (Hep G2, SK-Hep1, Huh7, HCCLM3, and MHCC97-H) validated the high expression of *RAB22A* in HCC cell lines (**Figure 1H**). The same results were obtained through qRT-PCR (*p <* 0.001) (**Figure 1K**). Next, we extracted 30 pairs of proteins from HCC and adjacent tissues and analyzed them using western blotting, which revealed that *RAB22A* was highly expressed in the former (**Figure 1I**). Results of western blot analysis of the liver and adjacent tissues are shown in **Supplementary Figure 1**. The high *RAB22A* mRNA expression levels in HCC tissues were further substantiated using qRT-PCR (*p* < 0.001) (**Figure 1L**). Immunohistochemistry (IHC) results also verified that *RAB22A* was upregulated in HCC tissues (**Figure 1J**). Finally, a receiver operating characteristic (ROC) curve was created. The ROC curve enclosed by the axes is the area below the curve (AUC). The AUC for *RAB22A* was 0.891, suggesting its remarkable diagnostic value for HCC (**Figure 1M**).

### 3.2 Association of RAB22A expression with clinical characteristics

**Figure 2:**
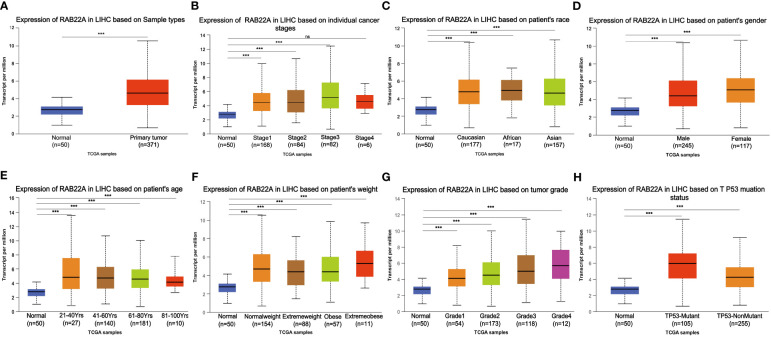
Box plot showing the relative transcription of RAB22A in individual cancer stages, race, gender, age, weight, tumor grade, and TP53 mutation status in a subgroup of patients with HCC. **(A)** *RAB22A* in normal and HCC tissues. **(B)** *RAB22A* in normal individuals or in patients with stages 1–4 liver cancer. **(C)** *RAB22A* in normal and LIHC samples based on patient ethnicity. **(D)** *RAB22A* in normal individuals and males and females with HCC. **(E)** *RAB22A* in healthy subjects of any age and patients aged 21–40, 41–60, 61–80, and 81–100 years with HCC. **(F)** *RAB22A* in healthy subjects of any weight and normal weight patients, extreme weight patients, obese patients, and extremely obese patients. **(G)** *RAB22A* in normal subjects and patients with different liver cancer tumor grades. **(H)** *RAB22A* in normal and TP53-mutant or TP53-non mutant patients. *p < 0.05, **p < 0.01, ***p < 0.001, NS, no significance.

Using the UALCAN database to perform subgroup analysis of numerous pathological characteristics, we found that *RAB22A* transcript levels were elevated in patients with HCC. (**Figure 2A**). The sub-group analysis of cancer stage, ethnicity, sex, age, weight, tumor grade, and TP53 mutation showed that the expression of RAB22A in HCC patients was significantly higher than that in the normal group (**Figures 2B–H**).

Logistic regression analysis showed that the increased expression of RAB22A in HCC was significantly correlated with sex (OR = 0.627 for male vs. female, *p =* 0.036), weight (OR = 0.567 for weight > 70kg vs. ≤ 70kg, *p* = 0.009), histological grades (OR=1.611 for G3 and G4 vs G1 and G2, *p* = 0.028), and tumor status (OR = 1.619 for with tumors vs. tumor free, *p* = 0.026). Conversely, RAB22A expression was not associated with age, M stage, T stage, N stage, height, BMI, AFP, or vascular invasion (**Table 1**).

**Table 1:** Association between *RAB22A* expression and clinicopathologic parameters by Logistic regression.

Characteristics	Total (N)	Odds Ratio (OR)	P value
Age (>60 vs. <=60)	373	0.851 (0.566-1.279)	0.438
M stage (M1 vs. M0)	272	2.912 (0.368-59.271)	0.357
**Gender (Male vs. Female)**	**374**	**0.627 (0.403-0.969)**	**0.036**
T stage (T3&T4 vs. T1&T2)	371	1.510 (0.942-2.437)	0.089
N stage (N1 vs. N0)	258	2.953 (0.373-60.136)	0.351
**Weight (>70 vs. <=70)**	**346**	**0.567 (0.369-0.867)**	**0.009**
Height (>=170 vs. < 170)	341	0.748 (0.484-1.152)	0.189
BMI (>25 vs. <=25)	337	0.758 (0.493-1.163)	0.205
AFP(ng/ml) (>400 vs. <=400)	280	1.608 (0.921-2.831)	0.096
Vascular invasion (No vs. Yes)	318	0.960 (0.604-1.526)	0.863
**Histologic grade (G3 & G4 vs. G1 & G2)**	**369**	**1.611 (1.053-2.475)**	**0.028**
**Tumor status (With tumor vs. Tumor free)**	**355**	**1.619 (1.061-2.478)**	**0.026**

The bold values indicates that the correlation analysis between RAB22A and clinicopathological parameters are statistically significant.

Next, we collected data from TCGA database to determine the clinicopathological parameters of *RAB22A* in different patients with HCC. Detailed information on the clinical data is provided in **Table 2**. After excluding cases without the necessary clinical data, 374 cases with a median age of 61.5 (range: 49.25−70.00) years and male preponderance of 67% were included. High expression of RAB22A in HCC was positively associated with tumor status (tumor-free vs. with tumor, *p* = 0.033), sex (female vs. male, *p* = 0.047), weight (≤ 70 vs. > 70, *p* = 0.012), and histological grade (grades 3 and 4 vs. grades 1 and 2, *p* = 0.031). These results indicate that the overexpression of RAB22A in HCC is closely related to the clinicopathological characteristics.

**Table 2:** Correlation between clinicopathological variables and *RAB22A* expression.

Characteristic	Low expression of *RAB22A*	High expression of *RAB22A*	P value
n	187	187	
T stage, n (%)			0.166
T1	96 (25.9%)	87 (23.5%)	
T2	49 (13.2%)	46 (12.4%)	
T3	36 (9.7%)	44 (11.9%)	
T4	3 (0.8%)	10 (2.7%)	
N stage, n (%)			0.622
N0	126 (48.8%)	128 (49.6%)	
N1	1 (0.4%)	3 (1.2%)	
M stage, n (%)			0.623
M0	132 (48.5%)	136 (50%)	
M1	1 (0.4%)	3 (1.1%)	
Pathologic stage, n (%)			0.293
Stage I	93 (26.6%)	80 (22.9%)	
Stage II	47 (13.4%)	40 (11.4%)	
Stage III	36 (10.3%)	49 (14%)	
Stage IV	2 (0.6%)	3 (0.9%)	
**Tumor status, n (%)**			**0.033**
Tumor free	110 (31%)	92 (25.9%)	
With tumor	65 (18.3%)	88 (24.8%)	
**Gender, n (%)**			**0.047**
Female	51 (13.6%)	70 (18.7%)	
Male	136 (36.4%)	117 (31.3%)	
Race, n (%)			0.940
Asian	79 (21.8%)	81 (22.4%)	
Black or African American	8 (2.2%)	9 (2.5%)	
White	88 (24.3%)	97 (26.8%)	
Age, n (%)			0.502
<=60	85 (22.8%)	92 (24.7%)	
>60	102 (27.3%)	94 (25.2%)	
**Weight, n (%)**			**0.012**
<=70	82 (23.7%)	102 (29.5%)	
>70	95 (27.5%)	67 (19.4%)	
Height, n (%)			0.228
< 170	96 (28.2%)	105 (30.8%)	
>=170	77 (22.6%)	63 (18.5%)	
BMI, n (%)			0.246
<=25	84 (24.9%)	93 (27.6%)	
>25	87 (25.8%)	73 (21.7%)	
Residual tumor, n (%)			0.217
R0	170 (49.3%)	157 (45.5%)	
R1	6 (1.7%)	11 (3.2%)	
R2	1 (0.3%)	0 (0%)	
**Histologic grade, n (%)**			**0.031**
G1	35 (9.5%)	20 (5.4%)	
G2	92 (24.9%)	86 (23.3%)	
G3	55 (14.9%)	69 (18.7%)	
G4	3 (0.8%)	9 (2.4%)	
AFP(ng/ml), n (%)			0.126
<=400	118 (42.1%)	97 (34.6%)	
>400	28 (10%)	37 (13.2%)	

The bold values indicates that the correlation analysis between RAB22A and clinicopathological parameters are statistically significant.

### 3.3 Prognostic value of *RAB22A* in HCC

**Figure 3:**
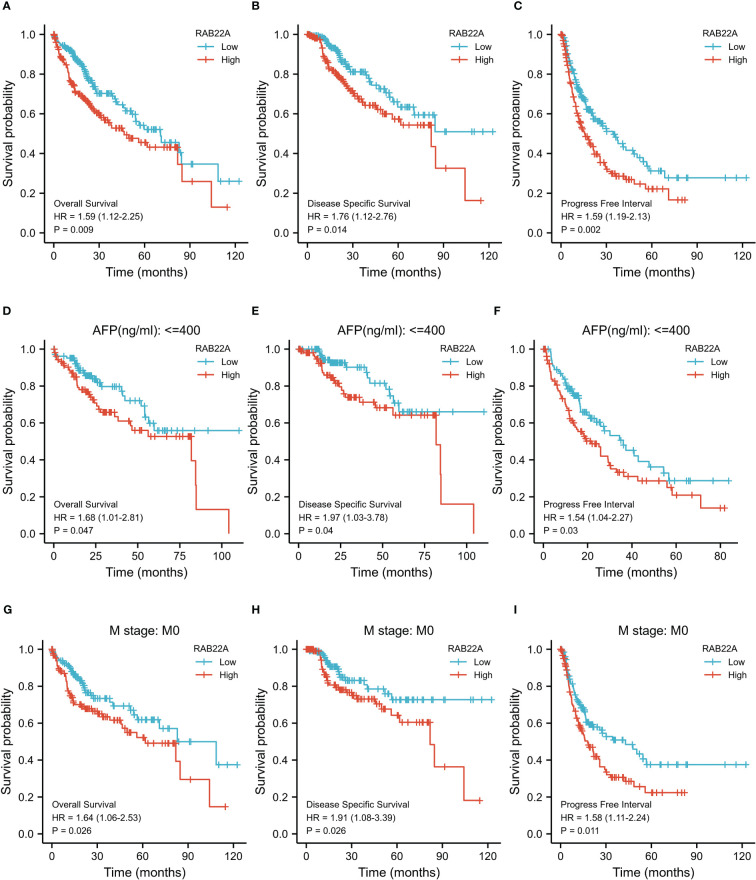
Kaplan–Meier survival plots comparing the relationship between *RAB22A* and prognosis in HCC. **(A–C)** Survival curves of OS, DSS, and PFI between RAB22A-high and -low patients with HCC. **(D–F)** OS, DSS, and PFI survival curves of patients with HCC with high and low *RAB22A* expression of AFP (ng/mL) ≤ 400. **(G–I)** Survival curves comparing OS, DSS, and PFI in patients with HCC at the M0 stage with high and low expression of *RAB22A*.

Kaplan–Meier survival curves were analyzed to determine the connection between *RAB22A* expression and overall survival (OS), disease-free survival (DSS), and progression-free interval (PFI) in the prognosis of patients with HCC. Increased levels of *RAB22A* expression were inversely related to prognosis (**Figures 3A–C**). Additionally, subgroup analysis was performed on patients with low RAB22A expression and AFP < 400, and these patients had better OS, DSS, and PFI prognosis (**Figures 3D–F**). However, the groups with AFP (ng/mL) > 400 showed no significant differences (**Supplementary Figures 2A–C**). The high expression of RAB22A in stage M0 liver cancer was associated with poor OS, DSS, and PFI in a subgroup of patients (**Figures 3G–I**). The subgroups of T3 versus T4, stages III vs. IV, and tumor versus tumor-free status had significantly worse OS (**Supplementary Figures 1D–F**). Finally, we compared predictive variables in patients with HCC obtained by univariate regression analysis to those obtained *via* multivariate survival analysis (OS) (**Supplementary Table 7**). Pathologic stage (stages I and II compared with stages III and IV; *p* <0.001), tumor size (T stages 1 and 2 versus T stages 3 and 4; *p* < 0.001), metastatic spread (M stages 0 and 1; *p* = 0.017), and tumor status (without or with tumor; *p <* 0.001) were highly significant in the univariate analysis. The multivariate analysis showed that with tumor (*p* = 0.014) was significant, suggesting that it is an independent risk factor.

### 3.4 GSEA and GO/KEGG enrichment analyses

**Figure 4:**
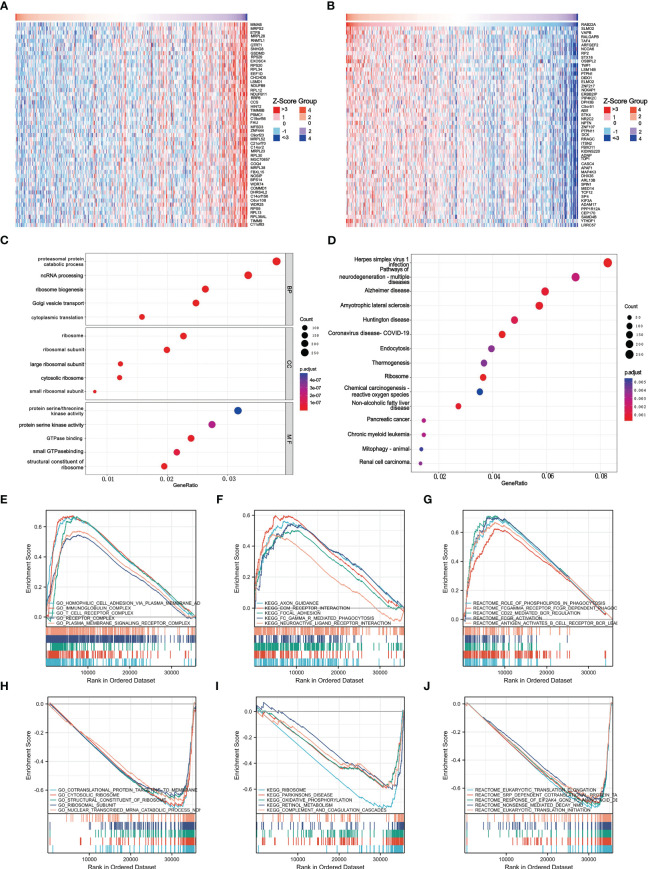
Enrichment of biofunction and associated gene analysis of *RAB22A* in HCC. **(A, B)** Heat map showing genes positively and negatively associated with *RAB22A* in liver cancer (top 50). Positively associated genes are indicated in red, while negatively associated genes are in green. **(C)** The enriched terms in GO categories in HCC. **(D)** KEGG pathway analysis based on *RAB22A*-associated DEGs. **(E)** The five most positively correlated pathways were revealed by GO term analysis. **(F)** KEGG pathway analysis revealed the five most positively correlated pathways. **(G)** The five most positively correlated pathways were identified *via* REACTOME pathway analysis. **(H)** The five most negatively correlated pathways were identified *via* GO term analysis. **(I)** KEGG pathway analysis identified the five most negatively correlated pathways. **(J)** The five most negatively correlated pathways were identified *via* REACTOME pathway analysis.

GO and KEGG pathway co-expression analyses of RAB22A-related genes in liver cancer mRNA sequencing data with 371 patients from the TCGA were performed using the functional module of Linkedomics. The top 50 marker genes and their connections with *RAB22A* expression are displayed on the heat map (**Figures 4A, B**; **Supplementary Table 8**). These findings revealed a widespread effect of *RAB22A* on the transcriptome.

Next, we conducted an enrichment analysis using the GO and KEGG databases to support the concept that *RAB22A*-related DEGs play a biological role in HCC (**Figures 4C, D**). The results of GO analysis showed that these DEGs were related to biological processes (BP), cellular components (CC), and molecular functions (MF). In the GO analysis, DEGs were enriched in diverse biological pathways, including proteasomal protein catabolic process, ncRNA processing, ribosome ribosomal subunit, protein serine/threonine kinase activity, and protein serine kinase activity. In the KEGG analysis, DEGs were highly concentrated in endocytosis and non-alcoholic fatty liver disease. GSEA was used to analyze the biological functions related to *RAB22A* expression.

The later criteria were enrichment score | NSE | > 1 (p < 0.05), according to which the five most positively relevant signal pathways were selected. GO analysis revealed that *RAB22A* expression was strongly positively correlated with the processes of homophilic cell adhesion *via* plasma membrane adhesion, immunoglobulin, T cell receptor, and plasma membrane signaling receptor complex (**Figure 4E**). The expression of *RAB22A* was inversely linked to that of co-translational proteins that bind to the membrane, the cytosolic ribosome, the structural components of the ribosome, the ribosomal subunit, and the nonsense-mediated decay of nuclear-transcribed mRNA catabolic processes (**Figure 4H**). KEGG analysis revealed that *RAB22A* expression was most strongly negatively connected with axon guidance, extracellular matrix receptor interaction, focal adhesion, FCγR-mediated phagocytosis, and the interaction with neuroactive ligand receptors (**Figure 4F**). The ribosome, Parkinson’s disease, retinol metabolism, oxidative phosphorylation, and complement and coagulation cascades were the top five most negatively correlated pathways (**Figure 4I**). REACTOME pathway analysis determined that phospholipids play a role in phagocytosis, Fc gamma receptor FCGR-dependent phagocytosis, CD22-mediated B cell receptor (BCR) regulation, and FCGR activation, and that second messenger is activated by BCR antigen that all positively correlated with RAB22A expression (**Figure 4G**). Eukaryotic translation initiation, eukaryotic translation elongation, translocation, the response of eukaryotic initiation factor 2 alpha subunit kappa B cyclin N2 to amino acid deprivation, co-translational protein of SRP-dependent targeting to the membrane, and nonsense-mediated decay were all negatively correlated with *RAB22A* expression (**Figure 4J**).

### 3.5 PPI network analysis

**Figure 5:**
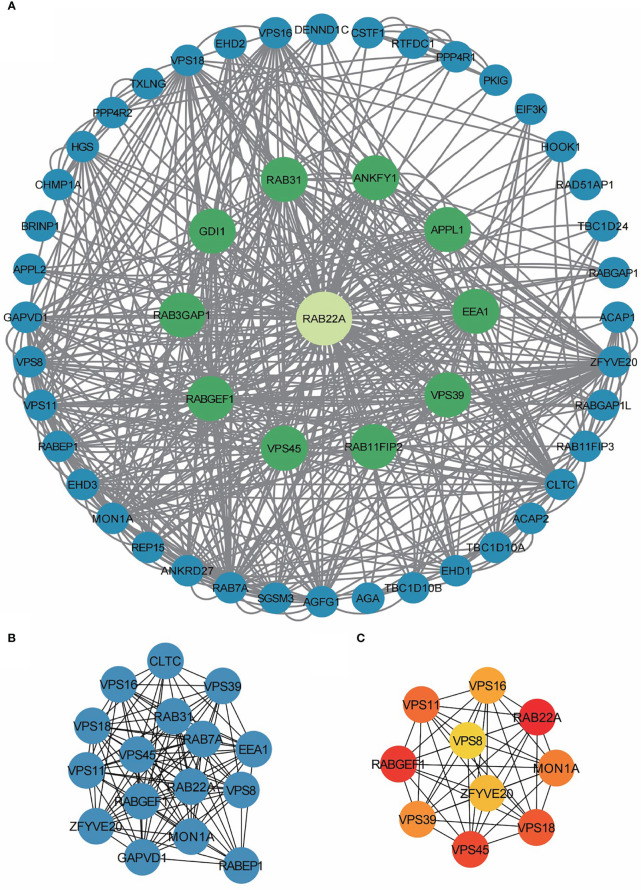
PPI network enrichment analysis. **(A)** The PPI network was built based on PPI pairs identified by the STRING dataset. **(B)** Hub gene clusters were selected from the PPI network (criteria of total scores ≥ 14,000). **(C)** Top 10 hub genes in the PPI network.

The PPI network of co-expressed genes conforming to the STRING conditions was assembled and visualized using Cytoscape, and analysis of the interactions among 108 DEGs in the HCC group was conducted. A total of 51 proteins and 534 edges were screened (**Figure 5A**; **Supplementary Table 9**).

After screening 12 nodes and 212 edges, a primary gene cluster with a total score ≥ 14,000 was discovered (**Figure 5B**). Finally, we screened the top 10 central genes, namely *RAB22A*, *RABGEF1, VPS45, VPS18, VPS11, MON1A, VPS39, VPS16, ZFYV20*, and *VPS8* (**Figure 5C**).

### 3.6 Role of RAB22A and m6A methylation regulators in HCC

**Figure 6:**
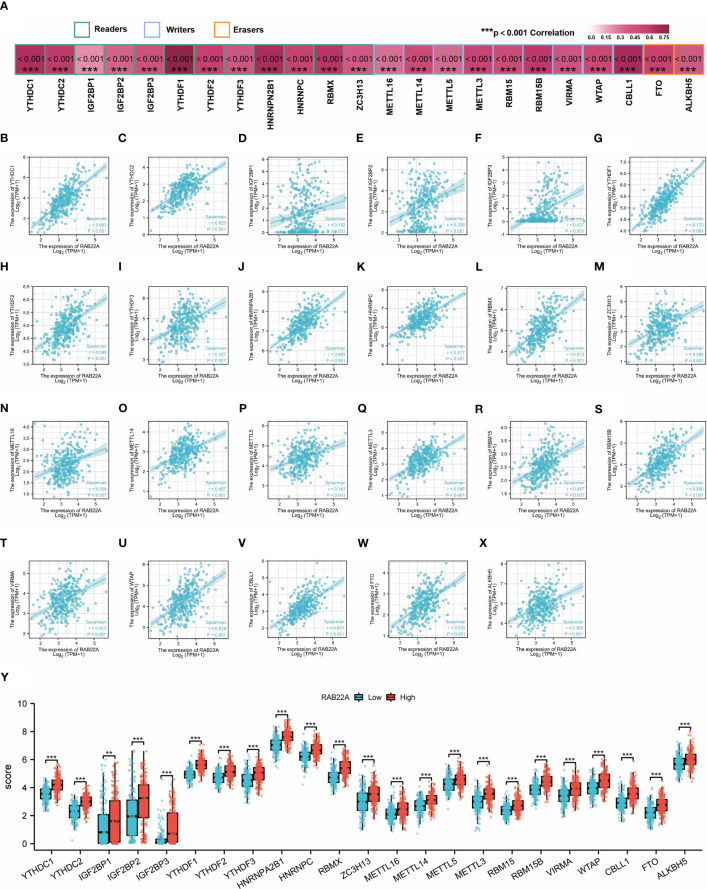
Correlation analysis of *RAB22A* expression levels with m6A-related gene expression in HCC tissues. **(A)** Correlation of *RAB22A* expression levels with m6A gene expression in HCC. **(B–X)** Scatter plot showing the relationship between *RAB22A* and the m6A gene. Differences in 23 M6A-related genes between the *RAB22A* high-expression group and *RAB22A* low-expression group in liver cancer patients **(Y)**.**p < 0.01, ***p < 0.001, NS, no significance.

M6A methylation affects the development of HCC (24–27). The expression of *RAB22A* was compared with that of the 23 M6A methylation genes reported in the literature to verify this conclusion (**Figure 6A**). *RAB22A* expression was closely connected with that of the 23 m6A-related genes in HCC (**Figures 6B–X**). Moreover, groups were formed according to *RAB22A* median expression. By analyzing the differences in the 23 m6A methylation genes in *RAB22A* between the high- and low-expression groups of patients with HCC, we observed that the expression levels of all genes in the *RAB22A* high-expression group were upregulated (**Figure 6Y**). Overall, we observed an obvious relationship between m6A methylation and RAB22A expression levels in HCC.

### 3.7 Construction of a triple regulatory network for *RAB22A*-associated ceRNA

**Figure 7:**
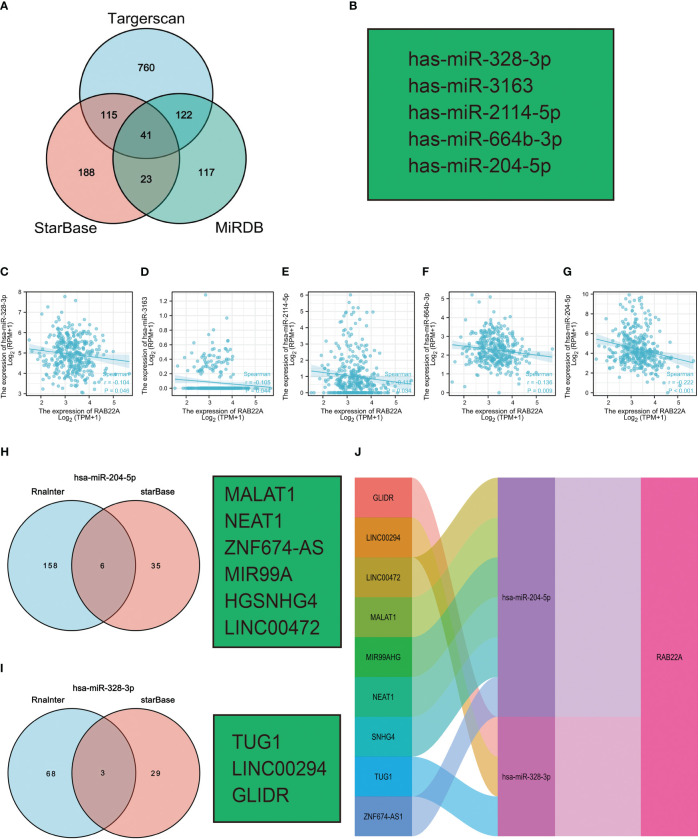
Prediction of ceRNA networks in HCC. **(A)** Venn diagram results showing 41 overlapping miRNAs in Targerscan, starBase, and MiRDB databases. **(B)** Five miRNAs screened for negative correlation with *RAB22A* expression. **(C–G)** Scatter plots showed that miRNAs were significantly correlated with mRNAs. **(H, I)** Prediction of lncRNAs bound to target miRNAs using miRNet and starBase online databases and displayed as a Venn diagram, including hsa-miR-204-5p and hsa-miR-328-3p. **(J)** Sankey diagram showing the *RAB22A*-related ceRNA regulatory network.

Increasing evidence has demonstrated the regulatory effect on the lncRNA-miRNA-mRNA ceRNA network in HCC. The Venn diagram showed 41 overlapping miRNAs in the Targerscan, starBase, and MiRDB databases (**Figure 7A**). Five human-derived miRNAs (miR-328-3p, miR-3163, miR-2114-5p, miR-664b-3p, and miR-204-5p) were verified to negatively correlate with *RAB22A* expression (**Figure 7B**). The expression of *RAB22A* and target microRNAs is displayed as a scatter plot (**Figures 7C–G**). We consulted the Rnalnter and starBase databases to predict lncRNAs that can have a mutual effect on target miRNAs (miR-204-5p and miR-328-3p) (**Figures 7H–I**). The expression levels of lncRNAs and miRNAs were inversely correlated, which accounted for the mutual influence between the two. We used the starBase database to filter and identify lncRNAs that were adversely associated with the two target miRNAs in HCC. Nine HCC-related ceRNA regulatory networks were constructed (**Figure 7J**).

### 3.8 Association of RAB22A expression with immune cell infiltration

**Figure 8:**
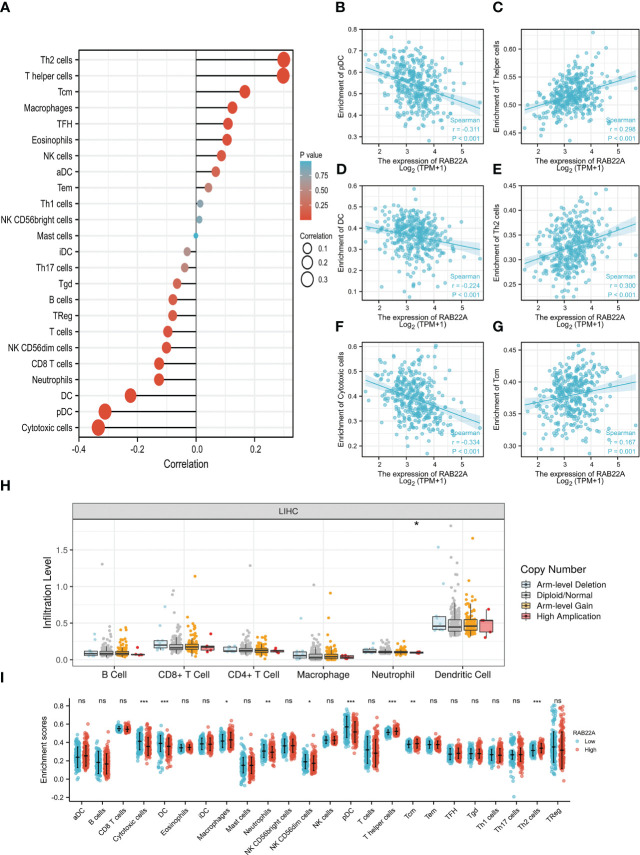
Relationship between the expression of RAB22A and microenvironment of immune infiltrating cells in HCC. **(A)** Forest plot depicting the relationship between *RAB22A* expression levels and the relative abundance of the 24 immune cells. **(B–G)** Scatter plots showing the degree of differentiation of pDCs, T helper cells, DCs, Th2 cells, cytotoxic cells, and Tcm cells between the high and low *RAB22A* expression groups. **(H)** SCNA showed that the expression of *RAB22A* correlated with the degree of immune cell infiltration. **(I)** Scatter plot showing the correlation of 24 immune cells with *RAB22A* expression levels. *p < 0.05, **p < 0.01, ***p < 0.001, NS, no significance.

Using the ssGSEA method, we verified the strong connection between *RAB22A* and immune cells (**Figure 8A**). The expression of *RAB22A* was positively connected with T helper cells, Tcm cells, and Th2 cells (*p* < 0.001) but negatively with cytotoxic cells, DCs, and pDCs (*p* < 0.001) (**Figures 8B–G**). RAB22A may be heavily involved in the T-cell immune response to HCC. Moreover, *RAB22A* expression in HCC correlated with various immune cell markers (**Table 3**). In the M2 macrophages in HCC, we found that *RAB22A* expression was substantially relevant to the expression of the immunological markers CD163, VSIG4, and MS4A4A. These results indicate that *RAB22A* caused the macrophages in HCC to adopt an M2 phenotype. The expression of *RAB22A* was substantially linked to 66 immunological markers, including CD8A, CD3D, and T-bet, in an analysis of functional T-cell immunity indicators. Furthermore, *RAB22A* expression was linked to immunological markers for B cells, T cells, TAMs, and neutrophils (**Table 3**). The TIMER database was utilized to determine whether *RAB22A* expression in HCC was connected with immune cell invasion levels. The results indicated that the CNV of *RAB22A* was related to the level of neutrophil infiltration. (**Figure 8H**). Subsequently, the infiltration of macrophages, T helper cells, Tcm, and Th2 cells increased (*p* < 0.001) in the *RAB22A* high-expression group; however, cytotoxic cells, DCs, and pDCs decreased (*p* < 0.001) (**Figure 8I**). These results verified that the increased expression of *RAB22A* in HCC is tightly linked with the infiltration of immune cells.

**Table 3:** Correlation analysis of *RAB22A* expression with immune cell biomarkers.

Description	Gene markers	LIHC	
		Cor	*P* -value
CD8+ T cell	**CD8A**	-0.463	**<0.001**
	**CD8B**	-0.418	**<0.001**
T cell (general)	**CD3D**	-0.446	**<0.001**
	**CD3E**	-0.561	**<0.001**
	**CD2**	-0.517	**<0.001**
B cell	**CD19**	-0.338	**<0.001**
	**CD79A**	-0.487	**<0.001**
Monocyte	**CD86**	-0.515	**<0.001**
	**CD115 (CSF1R)**	-0.530	**<0.001**
TAM	**CCL2**	-0.525	**<0.001**
	**CD68**	-0.440	**<0.001**
	**IL10**	-0.472	**<0.001**
M1 Macrophage	INOS (NOS2)	-0.089	0.099
	IRF5	0.003	0.962
	**COX2 (PTGS2)**	-0.501	**<0.001**
M2 Macrophage	**CD163**	-0.480	**<0.001**
	**VSIG4**	-0.488	**<0.001**
	**MS4A4A**	-0.512	**<0.001**
Neutrophils	**CD66b (CEACAM8)**	-0.106	**0.049**
	**CD11b (ITGAM)**	-0.330	**<0.001**
	**CCR7**	-0.552	**<0.001**
Natural killer cell	KIR2DL1	-0.043	0.422
	**KIR2DL3**	-0.184	**<0.001**
	**KIR2DL4**	-0.186	**<0.001**
	KIR3DL1	-0.105	0.050
	**KIR3DL2**	-0.221	**<0.001**
	KIR3DL3	-0.050	0.357
	KIR2DS4	-0.036	0.510
	**HLA-DPB1**	-0.490	**<0.001**
	**HLA-DQB1**	-0.454	**<0.001**
	**HLA-DRA**	-0.480	**<0.001**
	**HLA-DPA1**	-0.485	**<0.001**
	**BDCA-1 (CD1C)**	-0.426	**<0.001**
Dendritic cell	**BDCA-4 (NRP1)**	-0.195	**<0.001**
	**CD11c (ITGAX)**	-0.330	**<0.001**
Th1	**T-bet (TBX21)**	-0.436	**<0.001**
	**STAT4**	-0.259	**<0.001**
	**STAT1**	-0.192	**<0.001**
	**IFN-g (IFNG)**	-0.296	**<0.001**
	**TNF-a (TNF)**	-0.431	**<0.001**
Th2	**GATA3**	-0.499	**<0.001**
	STAT6	-0.003	0.957
	**STAT5A**	-0.250	**<0.001**
	IL13	-0.013	0.813
Tfh	BCL6	-0.009	0.866
	**IL21**	-0.160	**0.003**
	**STAT3**	-0.233	**<0.001**
	IL17A	-0.040	0.457
Th17	**FOXP3**	-0.226	**<0.001**
	**CCR8**	-0.320	**<0.001**
	**STAT5B**	0.162	**0.003**
	**TGFb (TGFB1)**	-0.410	**<0.001**
T cell exhaustion	**PD-1 (PDCD1)**	-0.429	**<0.001**
	**CTLA4**	-0.413	**<0.001**
	**LAG3**	-0.234	**<0.001**
	**TIM-3 (HAVCR2)**	-0.512	**<0.001**
	**GZMB**	-0.345	**<0.001**
Treg	**FOXP3**	-0.226	**<0.001**

The bold values indicates that the correlation analysis between RAB22A and biomarker of immune cell is statistically significant.

## 4 Discussion

*RAB22A* is a member of the RAS oncogene family that controls membrane properties and vesicle budding, delamination, movement, and fusion and is central to ensuring that cargo is transported to its correct destination. *RAB22A* is referred to in the early formation of endosomes and regulates vesicle transport (28, 29).

Furthermore, *RAB22A* is a critical oncogene that has a crucial impact on the course of many different forms of cancer (12, 30). *RAB22A* promotes the epithelial–mesenchymal transition of papillary thyroid cancer cells, thereby promoting their proliferation, migration, and invasion (31). CD147 is recycled by *RAB22A* to control lung carcinoma cell motility and invasion (13). In metastatic breast cancer, hypoxia facilitates MV production and HIF-dependent RAB22A gene expression (14). In addition, *RAB22A* is involved in a miRNA downregulation mechanism in which the overexpression of small GTPases promotes tumor growth and carcinogenesis. Several tumor models, including kidney, colorectal, glioma, and bile duct cancer, have utilized *RAB22A* as a target gene for miRNAs (32–34). Changes in *RAB22A* in HCC may be significant as hepatocytes always maintain high metabolic levels and active vesicular transport; nevertheless, the potential effect on *RAB22A* in HCC is unclear.

In the present study, we first found that *RAB22A* was upregulated in HCC and various malignant tumors by analyzing multiple databases. Subsequently, we verified the elevation of *RAB22A* expression in HCC cell lines and HCC samples using western blotting, qRT-PCR, and IHC *in vitro*. Overexpression of *RAB22A* in HCC tissues was closely associated with clinicopathologic features. The ROC curve analysis suggested RAB22A as a promising diagnostic biomarker for differentiating HCC from normal tissues. Moreover, the overexpression of *RAB22A* was interrelated with a poor prognosis of HCC, as indicated by OS, DSS, and PFI.

To elucidate the potential biological functions and regulatory pathways of *RAB22A*, we investigated genes encoding *RAB22A*-related proteins and co-expression genes in HCC tissues. mRNA sequencing data with HCC were evaluated in the TCGA database, while the DEGs associated with *RAB22A* in HCC were shown in a heat map. Insights gained from pathway enrichment analyses using GO and KEGG indicated that *RAB22A* has far-reaching effects on the transcriptome. Through enrichment pathway analysis, we verified that these DEGs were involved in proteasomal protein catabolic process, ncRNA processing, ribosomes, and ribosomal subunits, protein serine/threonine kinase activity, GTPase combining, herpes simplex virus type 1 infection, multiple neurodegenerative illnesses, and Alzheimer’s disease pathways. Next, we analyzed 30 signaling pathways positively and negatively correlated with *RAB22A* expression using GSEA. Overexpression of *RAB22A* was linked to processes such as cell adhesion *via* the plasma membrane (35), nonsense-mediated decay of nuclear-transcribed mRNA (36), axon guidance (37), oxidative phosphorylation (38), FCGR activation (39), and eukaryotic translation elongation (40) in a GSEA of HCC. Overall, we suggest that *RAB22A* may participate in various cellular immune functions and intracellular transport and may facilitate the advance of HCC by adjusting these signaling pathways.

Subsequently, we built a PPI network using Cytoscape. One central gene cluster (total score ≥ 14,000) and the top 10 central genes were filtered, namely *RAB22A*, *RABGEF1*, *VPS45*, *VPS18*, *VPS11*, *MON1A*, *VPS39*, *VPS16*, *ZFYV20*, and *VPS*. These findings provide important insights for subsequent study designs and experimental validations.

M6A methylation has been examined to elucidate the mechanisms of HCC since it has been proven to affect cancer *via* numerous mechanisms (41). m6A is a critical player in HCC (42, 43). Methyltransferases (the “Writers”), demethylases (the “Erasers”), and methylated reading proteins have access to the same m6A methylation (Readers). Methylation transferases, such as METTL3/14, WTAP, and KIAA1429, are primarily responsible for catalyzing the m6A alteration of adenosine on mRNA. Demethylases, such as FTO and ALKHB5, facilitate the demethylation of m6A. Methylation reading proteins, such as YTHDF 1-3 and YTHDC 1-3, recognize RNA methylation and play a role in regulatory processes, such as RNA translation, degradation, and miRNA processing (44). Further analysis of the connection between RAB22A expression and m6A methylation proteins revealed a positive and significant association between *RAB22A* expression and the expression of methylation transferases, demethylases, and methylated reading proteins. Patients with HCC have a poor prognosis because m6A-modified proteins are highly elevated in the disease, and their overexpression increases the disease progression. Several reports have verified that IGF2BP1, YTHDF1, and RBM15 are all highly elevated in HCC and contribute to its development and progression. These findings indicate that m6A may alter the *RAB22A* gene to enhance the consistency of its mRNA, hence boosting the occurrence and development of HCC. Evidence for lncRNA-miRNA-mRNA ceRNA networks’ regulatory role in cancers is mounting (45). Based on these predictions, we constructed a ceRNA regulatory network that predicted that *RAB22A* might affect several critical pathways of HCC regulatory mechanisms. We intend to conduct further experiments to validate this network.

Cancer cells that invade Immune cells, known as tumor-infiltrating immune cells (TIICs), play a key regulatory role in tumorigenesis and development (46). The HCC prognosis may be affected by the presence of TIICs, which are essential for HCC development (28–30). TIICs facilitate a tangled web of cellular interactions that boost the immunosuppressive milieu, facilitate immune escape, and ultimately aid in tumor progression. Changes in the immune environment of the liver can cause liver lesions, such as chronic inflammation and fibrosis/cirrhosis (22, 47). *RAB22A* is a regulator of immune functions. Independent studies have also shown that Th2 cells contribute to cancer development and progression (48, 49). Effector T helper cell subgroups are essential for coordinating immune responses to diverse infections and participate in the nosogenesis of numerous inflammatory disorders, including autoimmunity and allergies (50). pDCs are a sentinel cell type that can test pathogen-derived nucleic acids and reactions *via* the rapid and significant production of type I interferons, primarily in autoimmune diseases, immune deficiencies, and cancer (51). Cytotoxic T cells and DCs are also essential effectors of antitumor immunity (40, 52). These findings suggest that RAB22A plays an indispensable role in regulating immune cell infiltration in HCC.

We also demonstrated that *RAB22A* expression was significantly correlated with 66 immune markers. These results indicate that the upregulation of *RAB22A* expression in HCC is linked to immune cell infiltration. Understanding the function of RAB22A in immune activation will help to facilitate future research using various immune cell types and animal models.

Although our study identified the molecular mechanism of *RAB22A* in HCC through bioinformatics analysis, there remain limitations. Firstly, to elucidate the effect of *RAB22A* on HCC, several subjective factors, such as the treatment details received by patients and follow-up, should be considered simultaneously. However, some experiments were conducted in different centers, thereby limiting the information or causing inconsistency in the public database, which led to some errors. Secondly, the number of patients with cancer in the experimental control group was different from that in the current study; hence accessional studies are needed to eliminate the error caused by sample offset.

Thirdly, multicenter investigations based on communal databases seek to compensate for the paucity of single-center studies. However, retrospective studies have drawbacks, including inconsistent interventions and a lack of data. Since this study is retrospective, prospective investigations should be undertaken to eliminate analytical bias. Based on previous verifications, the results are robust; advancements in single-cell and spatial transcriptomics technologies allowed for the increased use of single-cell multi-omics technologies to gain insights into complex cellular ecosystems and biological processes. Currently, there is a gap in the rapidly growing single-cell multi-omics data, while effective methods for comprehensive analysis of these inherently sparse and heterogeneous data are limited. Therefore, new algorithms, such as SMGR (53) and spaCI (54), have been derived to address this gap. Single-cell multi-omics gene co-regulation algorithms provide multiple regulatory stages to study the control of cellular heterogeneity and complex biological mechanisms, which provide great clinical value for identifying mechanisms, targets, and predictors to enhance translational therapy. The spaCI algorithm can detect upstream transcription factors (TFS) mediating the L-R signaling axis, which provides insights into the underlying molecular mechanisms of the intercellular crosstalk. These emerging algorithms can be used to verify the biological mechanism of RAB22A in HCC.

In summary, we demonstrated, to the best of our knowledge, for the first time that *RAB22A* promotes carcinogenesis *via* m6A methylation and ceRNA network processes and is strongly linked with HCC development, poor survival, and immune infiltration.

## Data availability statement

The article/**Supplementary material** contains the original contributions presented in the study. Any additional questions can be forwarded to the corresponding authors. The datasets presented in this study can be found in online repositories. The names of the repository can be found below: https://portal.gdc.cancer.gov.

## Ethics statement

The studies involving human participants were reviewed and approved by First Affiliated Hospital of Harbin Medical University’s ethics committee. The patients/participants provided their written informed consent to participate in this study.

## Author contributions

FW, FM, XL, QL, and LJ contributed equally to this work. ZL and YC designed this research. FW and FM drafted this manuscript. XL , XW and QL performed the data collection and analysis. JL, RZ, YunZ, YuZ and SJ participated in the data interpretation and study design. All authors approved the final manuscript.
