# Supplementary material for: Characterization of prognostic value and immunological roles of RAB22A in hepatocellular carcinoma
Source: Front Immunol. 2023 Mar 3;14:1086342. doi: 10.3389/fimmu.2023.1086342 (PMC10021109; doi:10.3389/fimmu.2023.1086342)
Supplement: Supplementary file 1 [file DataSheet_1.docx]

Supplementary Material

Characterization of Prognostic Value and Immunological Roles of RAB22A in Hepatocellular Carcinoma

Fukai Wen^1,2†^, Fanshuai Meng^1,2†^, Xuewen Li^3†^, Qingyu Li^1,2†^, Jiaming Liu^1†^，Rui zhang^1,2^, Yunzheng Zhao^1,2^，Yu Zhang^1,2^，Xin Wang^1,2^ ，Shuai Ju^1,3^, Yifeng Cui^1,2*^, and Zhaoyang Lu^1,2*^

*** Correspondence:** Yifeng Cui: [cui88963342@hrbmu.edu.cn](mailto:cui88963342@hrbmu.edu.cn); Zhaoyang Lu: [lzy76772005@163.com](mailto:lzy76772005@163.com)

# Supplementary Methods

## Database Analysis and Processing

Gene expression profiles and related clinical data of 374 patients with HCC and 50 normal liver samples were collected from TCGA (https://portal.gdc.cancer.gov/) (Supplementary Table 1). RNA-seq data (TPM) were obtained together with information on the pertinent clinical aspects of the disease for patients with HCC. UALCAN, an interactive web portal comprising TCGA level 3 RNA-seq and clinical data from 33 cancer types, was used to analyze TCGA gene expression data in more depth. The UALCAN database (http://ualcan.path.uab.edu/index.html) was used to compare RAB22A transcription levels between HCC and normal liver samples as well as between diverse subtypes and substages. The datasets GSE121248, GSE76427, GSE87630, GSE84005, GSE54503, and GSE39791 were obtained from the GEO database (https://www.ncbi.nlm.nih.gov/geo)(17-19).

## Cell Lines and Cell Culture

The normal human liver cells (L02) and HCC cell lines (Huh7, HCCLM3, HepG2, Sk-Hep-1, and MCCH97-H) were obtained from the Chinese Academy of Sciences (Shanghai, China). L02 cells were cultured in RPMI-1640 medium. HCC cell lines were cultured in Dulbecco's modified Eagle's medium (DMEM), containing 10% fetal bovine serum (FBS) and 1% penicillin-streptomycin, and incubated at 37 °C in 5% CO_2_.

## Patients and Clinical Samples

Between February and June 2022, the First Affiliated Hospital of Harbin Medical University provided us with 30 matched sets of HCC and surrounding non-tumor liver tissues from patients undergoing hepatectomy. The Ethics Committee of the First Affiliated Hospital of Harbin Medical University approved this study. All patients provided written informed consent to participate in the study.

## Western Blotting

We extracted proteins from cells via radioimmunoprecipitation (RIPA) and separated them via electrophoresis (Beyotime, Shanghai, China). We transferred 300 mA of protein onto NC membranes (Millipore, Billerica, mA, USA) for 100 min. The membranes were blocked with 5% skim milk for 2 h and incubated with the primary antibody overnight at 4 °C, followed by incubation with the secondary antibody for 2 h. Protein visualization and analysis were performed using the Odyssey ® Imaging System (LI-COR, USA). Antibodies against RAB22A were purchased from Abcam (USA); secondary IgG anti-rabbit and mouse antibodies were also used.

## Quantitative reverse transcription-PCR (RT-qPCR)

Total RNA was extracted using TRIzol reagent and treated with DNase I to degrade any remaining DNA (Ambion). RT-qPCR was carried out on an ABI Prism 7900HT equipment (Life Technologies, Carlsbad, CA, USA) using the Power SYBR Green PCR master kit. The primers used are listed in Supplementary Table 2. The 2-DDCT approach was used to analyze the relative expression levels of *GAPDH* and other mRNAs to determine significant changes.

## Immunohistochemical (IHC) Analysis

Following deparaffinization in xylene and rehydration in a gradient ethanol series, antigen retrieval was carried out in an antigen-unmasking solution on tissue sections that had been formalin-fixed and paraffin-embedded (citrate-based).

The sections were first blocked with normal bovine serum and then incubated with optimally diluted primary antibodies (overnight at 4 °C), followed by biotinylated secondary antibodies (Vector Laboratories, Burlingame, CA, USA). Finally, to visualize immunoreaction products, the sections were counterstained with hematoxylin (Sigma, St. Louis, MO, USA) and stained with diaminobenzidine (DAB Kit; Vector Laboratories). Staining density was assessed using a previously described IHC procedure.(34)

## Tumor Immune Infiltration Analysis

The relative tumor infiltration levels of 24 immune cell types were quantified using single-sample gene set enrichment analysis (ssGSEA) to determine the expression levels of genes in published signature gene lists, while Spearman correlation and Wilcoxon tests were used to evaluate the correlation between RAB22A expression and immune cell infiltration in different groups. TIMER (https://cistrome.shinyapps.io/timer/) is a comprehensive resource for the analysis of tumor-immune interactions, including 10,897 samples of 32 types of cancer, and uses a deconvolution algorithm to infer the abundance of tumor-infiltrating immune cells (TIICs) from gene expression profiles; the correlation between the copy number variation (CNV) of RAB22A and the abundance of six types of TIICs (including B cells, CD4+ T cells, CD8+ T cells, neutrophils, macrophages, and dendritic cells) was explored. A correlation module was used to evaluate the correlation between RAB22A and immune cell marker gene expression. These genes are available online.

## Gene Set Enrichment Analysis

The LinkedOmics database (http://www.linkedomics.org) was selected to analyze multi-omics data and clinical data from 32 cancer types and 11,158 patients. The LinkFinder module of LinkedOmics showed that differentially expressed genes (DEGs) were in correlation with RAB22A in the LIHC cohort (n = 371, ID-124323). All results were analyzed using Pearson’s correlation coefficient and were demonstrated in the form of heat maps. The LinkInterpreter module identiﬁed the pathways and networks composed of DEGs. The data from the LinkFinder were signed and ranked, and GSEA was performed to elucidate GO (CC, BP, and MF) and KEGG pathways. The rank criterion was FDR < 0.05, and 500 simulations were performed.

GO term analysis, KEGG pathway analysis, and gene set enrichment analysis (GSEA) were performed to elucidate the biological function of RAB22A expression, with an enrichment score | NSE | > 1 (p < 0.05), and the five most relevant signal pathways were selected. The c5.all.v7.2. symbols.gmt, and c2.cp.v7.2. symbols.gmt curated gene sets were retrieved from the Molecular Signatures Database (MSigDB). A permutation test was performed 1,000 times to identify the significantly altered pathways. Adjusted p < 0.05 and FDR < 0.25 indicated significantly related genes. Pathway enrichment analyses were performed with the “clusterProfiler” R package.

## PPI Network and Module Analyses

The Search Tool for the Retrieval of Interacting Genes (STRING) (https://cn.string-db.org/) database of predicted functional associations between proteins contains 261,033 orthologs in 89 fully sequenced genomes. The co-expressed genes from the cBioPortal database were achieved and entered into STRING to construct the PPI network. The STRING database was utilized to analyze the interactions among 108 DEGs in the HCC group, and the interaction threshold was set to 0.15 to establish the PPI network. A total of 51 proteins and 534 edges were screened. The results were visualized using the Cytoscape software. Thereafter, the two most important modules were selected and subjected to functional enrichment analysis.

## Prediction and Construction of ceRNA Networks

TargetScan (http://www.targetscan.org), MiRDB (http://www.mirdb.org/), and StarBase (https://starbase.sysu.edu.cn/) online sites were used to predict and analyze the target miRNAs of RAB22A, compare the correlations between the expression of RAB22A and target miRNAs, and screen the miRNAs that were more compatible with ceRNA networks. The target lncRNAs of the screened miRNAs were predicted and analyzed using RNAinter (http://www.rnainter.org) and StarBase (www.starbase.sysu.edu.cn), and the correlation between the two was further analyzed to screen for additional eligible ceRNAs. A comprehensive analysis of negatively correlated miRNA–mRNA and miRNA-lncRNA expression levels was performed to establish an HCC-related lncRNA-miRNA-mRNA (RAB22A) ceRNA network.

# Supplementary Figures and Tables

## Supplementary Figures

**
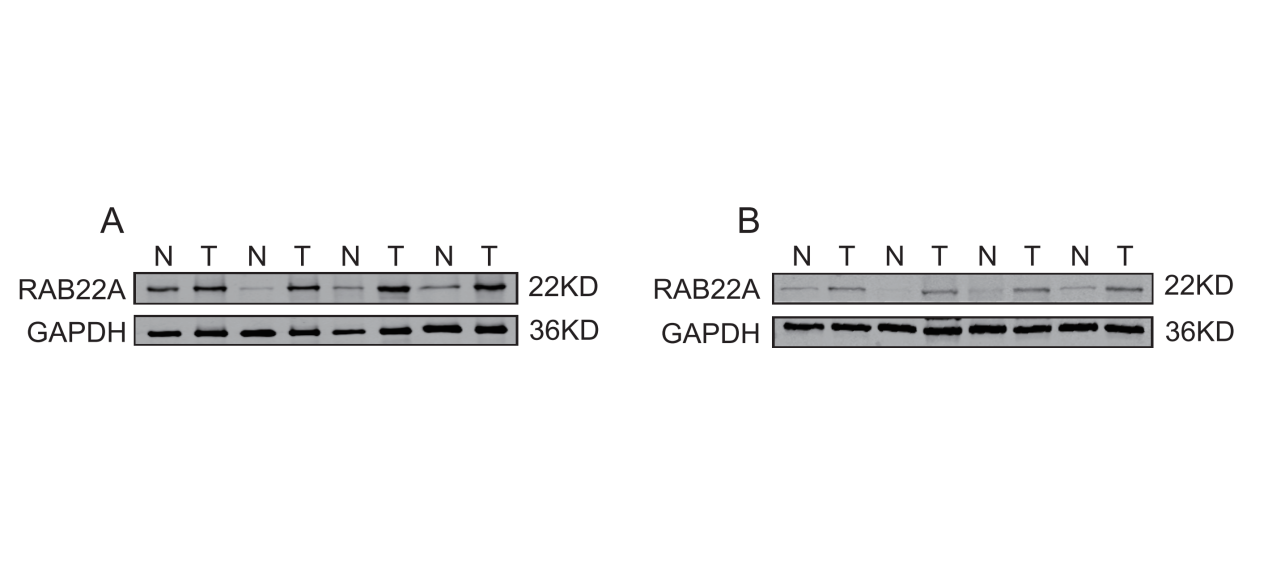
**

**Supplementary Figure 1** (A–B) Western blot assay of *RAB22A* protein expression levels in HCC and adjacent tissues.

**
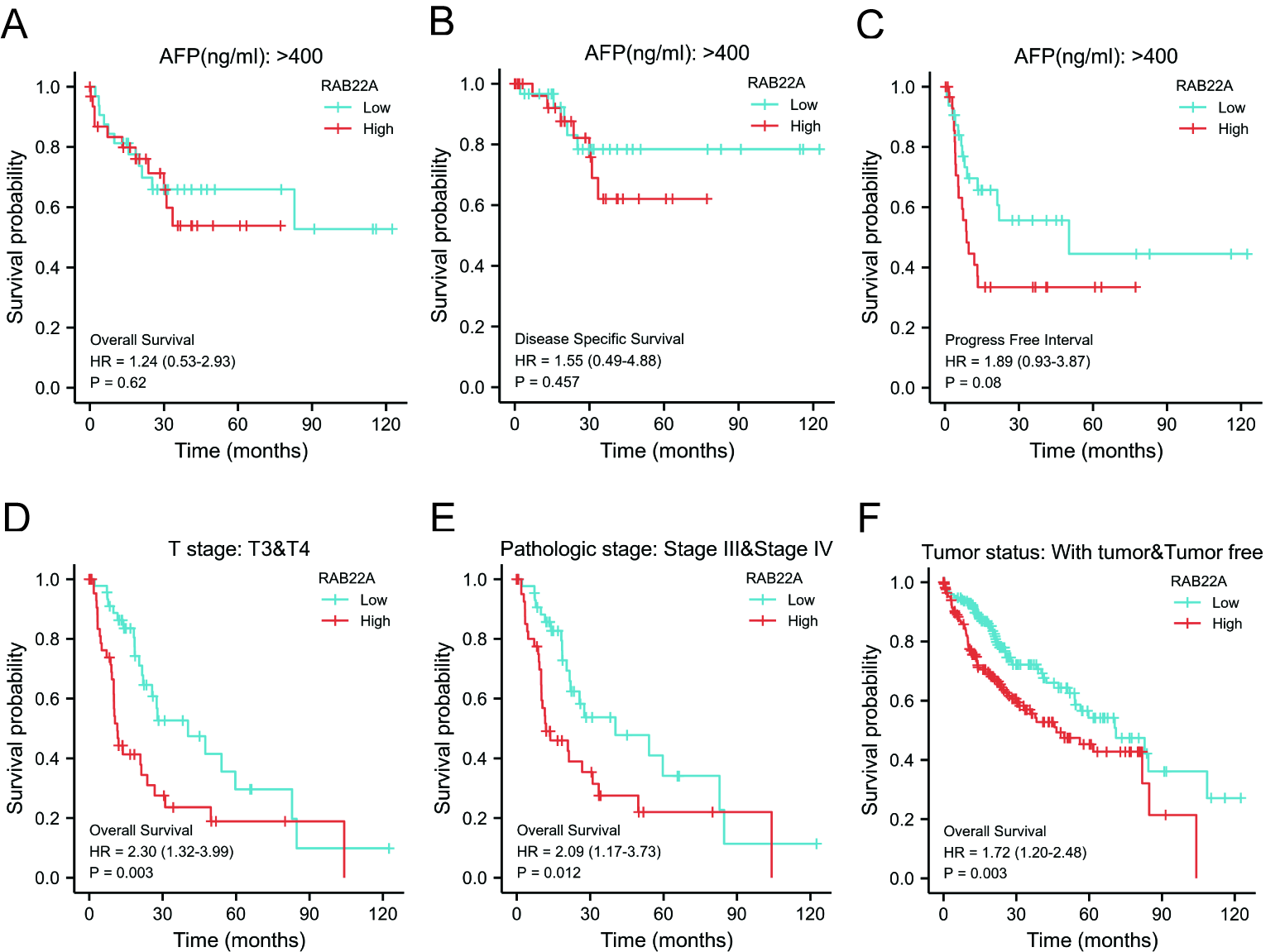
**

**Supplementary Figure 2.** Prognostic value of RAB22A in HCC. (A-C) OS, DSS, and PFI survival curves for AFP (ng/mL) ≤ 400 presenting with high and low RAB22A expression. (D) OS curves for patients with stage T3 and T4 HCC presenting with high and low RAB22A expression. (E) OS curves for patients with pathological stage III and IV HCC presenting with high and low RAB22A expression. (F) OS curves of HCC patients with tumor and tumor free with high and low expression of RAB22A.

## Supplementary Tables

**Supplementary Table 1.** Characteristics of patients with HCC in TCGA.

**Supplementary Table 2.** PCR primer sequence

**Supplementary Table 3.** GO enrichment analysis of RAB22A.

**Supplementary Table 4.** KEGG enrichment analysis of RAB22A.

**Supplementary Tables 5,6** GSEA enrichment analysis of RAB22A

**Supplementary Table 7.** OS of patients with HCC based on prognostic covariates.

**Supplementary Table 8.** Gene co-expression analysis of RAB22A.

**Supplementary Table 9.** PPI network of RAB22A.
