# Supplementary material for: Characterization of prognostic value and immunological roles of RAB22A in hepatocellular carcinoma
Source: Front Immunol. 2023 Mar 3;14:1086342. doi: 10.3389/fimmu.2023.1086342 (PMC10021109; doi:10.3389/fimmu.2023.1086342)
Supplement: Supplementary file 3 [file Table_2.docx]

| Gene | Direction | Sequence（5’-3’） |
| --- | --- | --- |
| RAB22A | Forward | GTGTGTCTGCTCGGGGATAC |
|  | Reverse | GCCCCTATTGTTGGGTTGATGT |
| GAPDH | Forward | TGACTTCAACAGCGACACCCA |
|  | Reverse | CACCCTGTTGCTGTAGCCAAA |
