# Supplementary material for: Characterization of prognostic value and immunological roles of RAB22A in hepatocellular carcinoma
Source: Front Immunol. 2023 Mar 3;14:1086342. doi: 10.3389/fimmu.2023.1086342 (PMC10021109; doi:10.3389/fimmu.2023.1086342)
Supplement: Supplementary file 8 [file Table_7.docx]

| Characteristics | Total(N) | Univariate analysis | |  | Multivariate analysis | |
| --- | --- | --- | --- | --- | --- | --- |
|  |  | Hazard ratio (95% CI) | P value |  | Hazard ratio (95% CI) | P value |
| Pathologic stage | 349 |  |  |  |  |  |
| Stage I&Stage II | 259 | Reference |  |  |  |  |
| Stage III&Stage IV | 90 | 2.504 (1.727-3.631) | **<0.001** |  | 1.452 (0.197-10.700) | 0.714 |
| T stage | 370 |  |  |  |  |  |
| T1&T2 | 277 | Reference |  |  |  |  |
| T3&T4 | 93 | 2.598 (1.826-3.697) | **<0.001** |  | 1.651 (0.223-12.219) | 0.623 |
| M stage | 272 |  |  |  |  |  |
| M0 | 268 | Reference |  |  |  |  |
| M1 | 4 | 4.077 (1.281-12.973) | **0.017** |  | 1.135 (0.268-4.796) | 0.864 |
| N stage | 258 |  |  |  |  |  |
| N0 | 254 | Reference |  |  |  |  |
| N1 | 4 | 2.029 (0.497-8.281) | 0.324 |  |  |  |
| Age | 373 |  |  |  |  |  |
| <=60 | 177 | Reference |  |  |  |  |
| >60 | 196 | 1.205 (0.850-1.708) | 0.295 |  |  |  |
| Gender | 373 |  |  |  |  |  |
| Female | 121 | Reference |  |  |  |  |
| Male | 252 | 0.793 (0.557-1.130) | 0.200 |  |  |  |
| Tumor status | 354 |  |  |  |  |  |
| Tumor free | 202 | Reference |  |  |  |  |
| With tumor | 152 | 2.317 (1.590-3.376) | **<0.001** |  | 1.824 (1.130-2.945) | **0.014** |
| Weight | 345 |  |  |  |  |  |
| <=70 | 184 | Reference |  |  |  |  |
| >70 | 161 | 0.941 (0.657-1.346) | 0.738 |  |  |  |
| BMI | 336 |  |  |  |  |  |
| <=25 | 177 | Reference |  |  |  |  |
| >25 | 159 | 0.798 (0.550-1.158) | 0.235 |  |  |  |
| Residual tumor | 344 |  |  |  |  |  |
| R0 | 326 | Reference |  |  |  |  |
| R1&R2 | 18 | 1.604 (0.812-3.169) | 0.174 |  |  |  |
| Histologic grade | 368 |  |  |  |  |  |
| G1&G2 | 233 | Reference |  |  |  |  |
| G3&G4 | 135 | 1.091 (0.761-1.564) | 0.636 |  |  |  |
| AFP(ng/ml) | 279 |  |  |  |  |  |
| <=400 | 215 | Reference |  |  |  |  |
| >400 | 64 | 1.075 (0.658-1.759) | 0.772 |  |  |  |
| RAB22A | 373 |  |  |  |  |  |
| Low | 187 | Reference |  |  |  |  |
| High | 186 | 1.588 (1.121-2.249) | **0.009** |  | 1.489 (0.928-2.389) | 0.099 |
